# Developing pharmacy leaders: A design thinking approach to navigate the leadership crisis in pharmacy

**DOI:** 10.1177/17151635241240470

**Published:** 2024-04-09

**Authors:** Kayla Byers, Angela Gee, Maria Anwar

**Affiliations:** Faculty of Pharmacy and Pharmaceutical Sciences, Alberta Health Services, Edmonton, Alberta; University of Alberta, Edmonton, Alberta; and Pharmacy Services, Alberta Health Services, Edmonton, Alberta; University of Alberta, Edmonton, Alberta; and Pharmacy Services, Alberta Health Services, Edmonton, Alberta

## Background

A quick search of the term *leadership* within the *Canadian Pharmacist Journal* reveals a decade-long discussion about the leadership crisis in pharmacy. Despite the issue emerging over a decade ago and gaining considerable attention, there remains a notable lack of interest and readiness among pharmacists to pursue leadership roles.^1^ As the pharmacy profession continues to evolve from a product-based service to a patient-centred practice, our leadership structure must adapt accordingly, because a profession lacking leaders from within is simply unsustainable.^2^ The question we confront is how to inspire motivation for leadership development among frontline pharmacists and help drive our profession forward.

To help address this crisis, this project used design thinking methodology to understand the perspective of frontline pharmacists and cocreate solutions tailored to their needs. The overarching goal was to foster meaningful engagement in leadership, with the insights of pharmacists guiding the way toward sustainable results. This approach recognized the complexity of the leadership deficit in pharmacy and the need for creative solutions.

## Approach

To address the complexity of leadership development in health care, a project team consisting of 2 pharmacy students and 2 clinical practice lead copreceptors applied a design thinking methodology. This methodology employs a nonlinear process that aims to redefine problems and create innovative solutions by incorporating the needs of the user into the solution.^3^ Involving frontline pharmacists in the solution design process allowed the team to reframe the unique challenge of leadership development in a human-centred way, with the goals and aspirations of pharmacists at the forefront. The project spanned 8 weeks in a suburban hospital setting in the southwest zone of Alberta Health Services (AHS) and received support from 2 senior leadership executives, who provided global insights aligned with organizational values.

**Figure 1:**
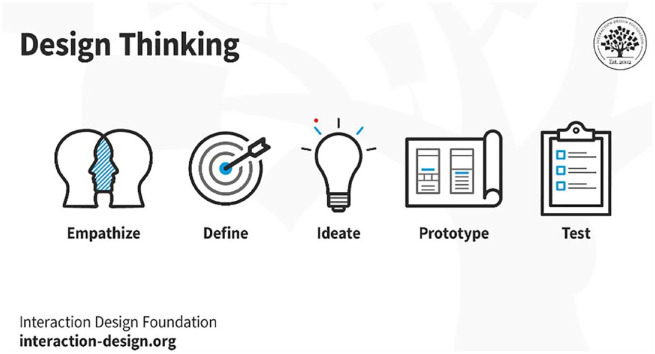
The 5 stages of design thinking

The design thinking approach consists of 5 stages: empathize, define, ideate, prototype and test (as illustrated in Figure 1).^3^ The empathize stage of this project consisted of a thorough exploration of the perspectives of frontline pharmacists, incorporating diverse methods such as a literature review, 1-on-2 interviews, surveys and focus groups. The insights gathered were used to clearly define the needs and challenges faced by frontline pharmacists concerning leadership development, with the collected data organized into emergent themes. Moving on to the ideate phase, the project team engaged in brainstorming creative solutions, subsequently creating and testing prototypes of targeted leadership development activities.

## Findings

Semi-structured, conversational interviews were conducted with 8 pharmacists from the Chinook Regional Hospital (CRH), providing valuable insights into their definition of leadership, key attributes, notable accomplishments, competencies and perceived benefits of leadership development. Postinterview surveys were used in conjunction to gauge how highly participants valued different leadership competencies, on a Likert scale of 1 (not valuable at all) to 10 (extremely valuable).

The definition of leadership identified by participants primarily revolved around concepts such as leading by example, advocating for roles and the ability to effect change. Key attributes deemed to be essential to effective leadership included approachability, adaptability, compassion, empathy, clinical expertise and problem-solving skills. Noteworthy accomplishments included resilience in managing chaos, successful completion of clinical projects and efforts toward standardization in clinical practice.

The most valued competencies for leadership development for frontline pharmacists in this project were lifelong learning, self-awareness and possessing a global perspective. Interestingly, leadership knowledge and business skills were less valued competencies among the group, highlighting the emphasis placed on personal development over formal training.

A common barrier for pharmacists developing leadership skills was an overwhelming desire to be a clinical expert in their area first before shifting their focus to leadership. This goal was similarly expressed by senior leadership, who ultimately want all pharmacists to provide the best possible patient care. Participants acknowledged that leadership development could provide benefits such as enhanced confidence, elevated patient care standards and a contribution to personal growth and collaboration. These benefits could extend beyond leadership development and into clinical practice. Thus, if we reframe the way we think about leadership development as a means to improve our individual and our team’s clinical practice, we can work towards our common goal of enhancing patient care.

The insights that emerged from these data informed the creation of leadership development activities, which were prototyped and tested among CRH pharmacists. The activities were focused on self-awareness, which was a competency valued highly by participants. One such activity was a “Leadership Development Portfolio” designed to explore personal values, skills and qualifications to track personal development, creating a portfolio to document accomplishments and enhance self-awareness. The prototyped activities subsequently inspired the creation of “Team Lead” positions as voluntary leadership roles that enable frontline pharmacists to develop and practise these desired skills. These positions are currently being employed in the CRH and may present a valuable solution to the leadership crisis.

## Discussion

The key findings regarding the definition of leadership among frontline pharmacists included leading by example, advocacy and the ability to effect change. The most valued competencies for leadership development were self-awareness and global perspective. This project found that the reluctance to take on leadership roles stemmed from the desire to first excel in a specific clinical area before shifting focus to leadership. It also highlighted the acknowledgment that leadership development could provide benefits beyond leadership roles and into clinical practice by improving confidence and collaboration.

There is literature that supports the correlation between leadership development and improvement to clinical care. A recent study examined the impact of leadership styles on health care quality and found a positive correlation between leadership at all levels and patient satisfaction, as reported by staff, patients and clinical measurements.^4^ These results reiterate the findings of this project, and demonstrate the impact that leadership development can have on the quality of patient care, confirming that leadership matters at all levels in health care.^4^

The development of leadership among frontline pharmacists aligns with a distributed leadership model that enables individuals to make decisions locally, guided by the organization’s overall goals, visions and mission.^5^ This model is crucial as the pharmacist profession continues to transform, necessitating mutual accountability and professional identity development.^5^ Improvements in clinical care happen when clinicians have an increased sense of accountability and professional identity.

The most valued competencies of leadership development, self-awareness, a global perspective and lifelong learning are intrinsically linked to personal growth and development. By developing skills such as increased confidence, effective communication, decision-making and problem-solving, pharmacists can simultaneously enhance their leadership capabilities and clinical practice to ultimately provide the best possible patient care.

## Implications

Engaging frontline pharmacists in design thinking methodology allows for the discovery of creative solutions to address the current pharmacy leadership crisis. The findings from this project offer insights into barriers to pursuing leadership development in pharmacy and sustainable, creative solutions to address these challenges. By listening to the perspectives of frontline pharmacists, creating tailored leadership development activities together and focusing on the benefits to patient care, we can simultaneously enhance leadership and quality of clinical practice in the best interest of patients. ■
